# Algorithm of rational approach to reconstruction in Fournier’s disease

**DOI:** 10.1515/med-2021-0294

**Published:** 2021-07-09

**Authors:** Domenico Tripodi, Maria Ida Amabile, Federica Gagliardi, Federico Frusone, Marzia Varanese, Alessandro De Luca, Daniele Pironi, Vito D’ Andrea, Salvatore Sorrenti, Claudio Cannistrà

**Affiliations:** Department of Surgical Sciences, Sapienza University of Rome, Rome, CAP 00161, Italy; Plastic and Reconstructive Surgery Unit, Centre Hospitalier Universitaire Bichat Claude-Bernard, Paris, France

**Keywords:** Fournier’s gangrene, inguinal flap, full-thickness skin graft, perineal reconstruction, reconstructive technique

## Abstract

**Background:**

Fournier’s gangrene is a rare form of necrotizing fasciitis that affects the genital area up to the perineal region and sometimes the abdominal wall.

**Objectives:**

Our article aims to show that in the treatment of extensive forms of Fournier’s gangrene, correct use of flap and skin grafts and a quick reconstruction of the exposed tissues avoid scarring retraction of the testicles and deformation of the penis.

**Materials and methods:**

We retrospectively reviewed the clinical and photographic data of Fournier’s gangrene cases treated at our Institute. The data were evaluated to obtain an estimate of the results of the reconstructive technique used, in terms of percentage of occurred healings and eventual complications.

**Results:**

A total of 34 patients underwent surgery for Fournier’s gangrene. In nine cases (26.5%), we had minor complications: in four patients, suffering from diabetes and obesity, a retard in attachment of graft occurred, while in five patients with perianal problems there was a delay in healing due to the onset of local infection.

**Conclusion:**

The reconstruction approach described here may reduce surgical times. In Fournier’s gangrene, the exposed tissues must be reconstructed as quickly as possible.

## 1 Introduction

Fournier’s gangrene is a rare form of necrotizing fasciitis that affects the genital area up to the perineal region and sometimes the abdominal wall. In advanced forms, the disease determines skin necrosis and sometimes deep tissue necrosis up to systemic complications [1]. This condition represents a surgical emergency and has a high mortality rate, ranging from 0 to 67% [2]. Several authors worked hard to determine the cause generating this inflammatory infectious process [3,4], and several triggers were identified as intravenous drug abuse [5], radiotherapy [6], diabetes [7], obesity [8], anal and urogenital disease [9], cancer, and iatrogenic factors [10,11]. It has been hypothesized that Fournier’s gangrene may be determined by a polymicrobial infection progressing to obliterative endarteritis with microthrombosis of the cutaneous and subcutaneous arterioles and consequent peripheral spread of bacteria, causing gangrene of the overlying tissues [12]. The bacterial culture of the interested tissue often allows to isolate multiple organisms, including anaerobes and aerobes, although the Fournier’s gangrene diagnosis is essentially clinical. Clinical features include sudden onset of genital or perineal pain with swelling of the tissues associated with fever and prostration. Treatment of this condition is based on the management of the underlying cause with anti-inflammatory drugs and targeted antibiotics, as well as reconstructive techniques for the loss of substance [13]. Reconstructive strategies include flaps [14,15], granulation for second intention or vacuum-assisted closure therapy [16], and skin graft [17]. Moreover, other surgical techniques have been described to repair the loss of substance in Fournier’s disease, consisting an oblique rectus abdominal myocutaneous flap, superiority of medial circumflex femoral artery perforator flap, and posteromedial thigh propeller flap [18,19,20]. The aim of our article is to document our 16-year experience in the treatment of extensive forms of gangrene of Fournier, emphasizing a correct use of flap and skin grafts.

## 2 Materials and methods

We retrospectively reviewed the clinical and photographic data of Fournier’s gangrene cases treated at our Institute from 2004 to 2020. The data collected includes past medical history, type of surgical reconstruction, surgical timing, medium follow-up, and photographic images. According to guidelines [21], the abdominal-pelvis CT scan was the investigation preferred in most cases for early diagnosis and/or atypical presentations to assess the extension of the disease and to reveal thickening of the scrotal wall and eventual presence of gases in the subcutaneous tissue. Moreover, imaging study with abdominal-pelvis ultrasound and in particular with magnetic resonance imaging was useful in those cases where a further pelvis diagnostic study was requested.

In collaboration with the Infectious Disease Unit, the considered patients were previously treated with antibiotic therapy and hospitalization in the intensive care unit when necessary and then addressed to our attention for the reconstruction phase. The data were evaluated to estimate the results of the reconstructive technique used, in terms of percentage of occurred healings, eventual complications, number of surgical reoperation, and percentage of affected body’s areas.


**Ethical approval:** All procedures performed in studies involving human participants were in accordance with the ethical standards of the institutional and/or national research committee and with the Declaration of Helsinki 1964 and its later amendments or comparable ethical standards.
**Informed consent:** Written informed consent for patient information and images was provided by a legally authorized representative. All the procedures performed in this study were in accordance with the Declaration of Helsinki 1964 and its later amendments or comparable ethical standards.

## 3 Results

**Figure 1:**
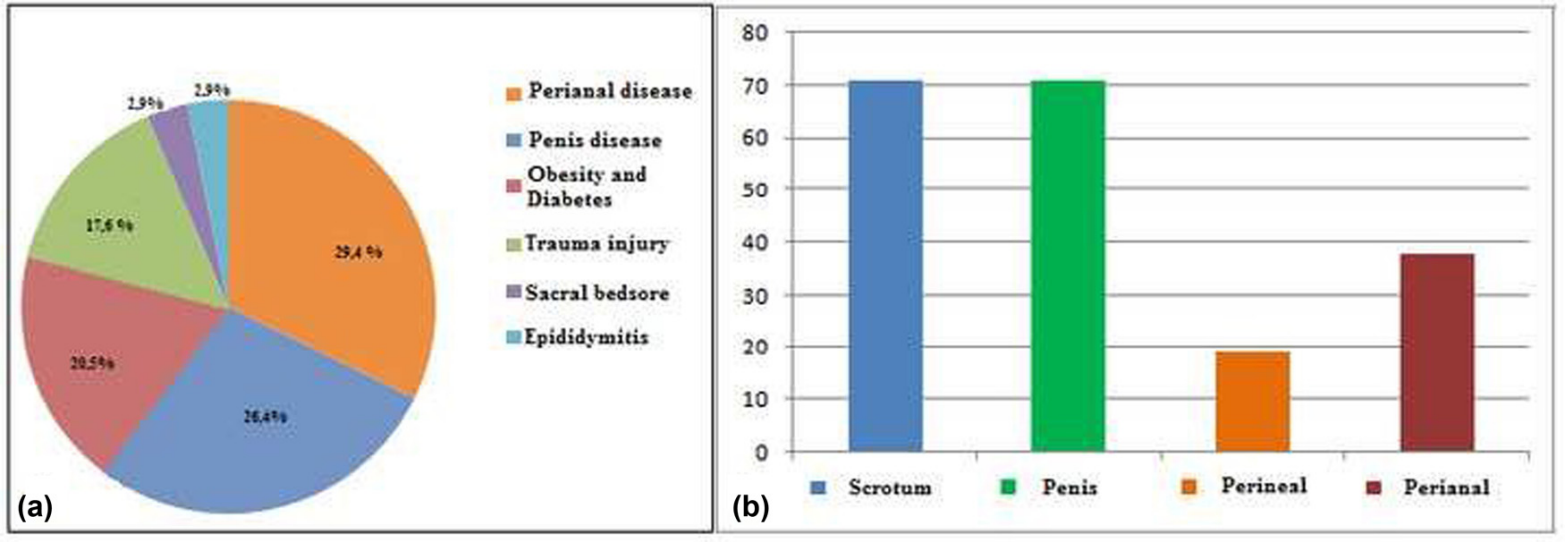
(a) Diseases observed at the origin of the infective process. (b) Percentage of the anatomical regions interested by the gangrene.

From 2004 to 2020, 34 patients underwent surgery for Fournier’s Gangrene at our Institution (Table 1). The average age was 47.9 years (range 20–72 years). As shown in Figure 1, in ten cases (29.4%) trigger points developed in the perianal area, while in nine cases (26.4%) the gangrene developed in penis tissue; obesity and decompensated diabetes were implicated in seven cases (20.5%), while for six patients (17.6%) a trauma injury was identified as the origin of the tissue gangrene; in one case (2.9%) the gangrene overlapped on sacral bedsores and in one case (2.9%) epididymitis episode was the trigger factor. In 24 cases (70.5%), we documented a scrotum involvement, while penis involvement occurred in other 24 cases (70.5%), in six cases (17.6%) perineal and in 13 cases (38.2%) perianal tissue involvement were observed. An extensive debridement was performed in all cases followed by reconstruction with thickness graft or proximity flap. For one patient’s testis, scrotum and glans direct amputation was performed, and in another case, penis amputation was necessary because of a concomitant advanced cancer. Only one patient (2.9%) underwent two surgical reconstructive sessions, the second one consisting in scrotal reconstruction with fascial-myocutaneous pedunculated free flap applied in inguinal fold performed 1 year later the first surgery session due to a progressive retraction of scrotum region. Mean follow-up was 7.5 months (range 5–18 months). In one case, the patient died during the hospitalization due to the aggravation of sepsis. In nine cases (26.5%), there were minor complications: four patients were suffering from diabetes and obesity with a delay in the engraftment of the graft occurred, while in five patients had perianal problems and there was a delay in the healing due to the onset of local infection. No cases of problems related to difficulty in penis erection and/or scar retraction were reported.

**Table 1:** Patients’ characteristics including number and type of surgery performed

Patient’s age (years)	Trigger factors	Reconstructive procedures				
		Scrotum	Penis	Perineal	Perianal	Operating session
48	Complication of diabetes and obesity	Time 1: Debridement and implant of the testicle in a medial thigh pocket	Time 1: Debridement and full-thickness graft from the upper arm		Time 1: Debridement and split thickness graft	1
30	Penile self-injection of vaseline		Time 1: Debridement and full-thickness graft from the upper arm			1
42	Penile self-injection of vaseline in a diabetic patient		Time 1: Debridement and full-thickness graft from the upper arm			1
64	Perianal abscess in a diabetic patient	Time 1: Contralateral proximity scrotal flap			Time 1: Proximity flap from the gluteal region	1
68	Perianal fistula in hepatic transplant patient	Time 1: Debridement and implant of the testicle in a medial thigh pocket			Time 1: Debridement and split thickness graft	1
32	Penile self-injection of vaseline		Time 1: Debridement and full-thickness graft from the upper arm			1
52	Trauma injury	Time 1: Debridement and groin flap	Time 1: Debridement and full-thickness graft from the upper arm			1
59	Diabetes and severe chronic renal failure	Time 1: Debridement and proximity scrotal flap	Time 1: Debridement and full-thickness graft from the upper arm			1
30	Complications of penile implants	Time 1: Debridement and proximity scrotal flap	Time 1: Debridement and full-thickness graft from the upper arm			1
49	Perianal fistula	Time 1: Debridement and contralateral proximity scrotal flap			Time 1: Debridement and split-thickness graft	1
51	Perianal abscess			Time 1: Debridement and split-thickness graft	Time 1: Debridement and split-thickness graft	1
33	Trauma injury	Time 1: Debridement and implant of the testicle in a medial thigh pocket	Time 1: Debridement and full-thickness graft from the upper arm	Time 1: Debridement and split-thickness graft	Time 1: Debridement and split-thickness graft	2
		Time 2: Groin flap				
58	Superinfection of hemorrhoids disease	Time 1: Debridement and implant of the testicle in a medial thigh pocket	Time 1: Debridement and full-thickness graft from the upper arm		Time 1: Debridement and split-thickness graft	1
65	Trauma injury	Time 1: Debridement and implant of the testicle in a medial thigh pocket	Time 1: Debridement and full-thickness graft from the upper arm	Time 1: Debridement and split-thickness graft		1
42	Epidermoid carcinoma of the penis		Time 1: Debridement and full thickness graft from the upper arm			1
43	Complication of diabetes and obesity	Time 1: Debridement and proximity scrotal flap	Time 1: Debridement and full-thickness graft from the upper arm			1
54	Trauma injury	Time 1: Debridement and contralateral proximity scrotal flap	Time 1: Debridement and full-thickness graft from the upper arm	Time 1: Debridement and split-thickness graft		1
63	Complication of diabetes and obesity	Time 1: Debridement and implant of the testicle in a medial thigh pocket	Time 1: Debridement and full-thickness graft from the upper arm			1
72	Perianal infection	Time 1: Debridement with testicles and scrotum amputation	Time 1: Debridement with glans amputation		Time 1: Debridement and split-thickness graft	1
50	Perianal infection				Time 1: Debridement and split-thickness graft	1
48	Penis cancer		Time 1: Penis amputation	Time 1: Debridement and split thickness graft		1
50	Epididymitis	Time 1: Debridement and implant of the testicle in a medial thigh pocket	Time 1: Debridement and full-thickness graft from the upper arm			1
52	Perianal infection	Time 1: Debridement and implant of the testicle in a medial thigh pocket	Time 1: Debridement and full-thickness graft from the upper arm		Time 1: Debridement and split-thickness graft	1
60	Perianal infection				Time 1: Debridement and split-thickness graft	1
50	Perianal infection	Time 1: Debridement and implant of the testicle in a medial thigh pocket			Time 1: Debridement and split-thickness graft	1
25	Complication of circumcision	Time 1: Debridement and proximity scrotal flap	Time 1: Debridement and full-thickness graft from the upper arm			1
29	Complication of sacral bedsore in paraplegic patient	Time 1: Debridement and implant of the testicles in a medial thigh pocket	Time 1: Debridement and full-thickness graft from the upper arm		Time 1: Debridement and split-thickness graft	1
50	Superinfection of second-degree burn of the testicles region	Time 1: Debridement and proximity scrotal flap	Time 1: Debridement and full-thickness graft from the upper arm			1
68	Complication of diabetes and obesity	Time 1: Debridement and proximity scrotal flap				1
42	Complication of diabetes and obesity	Time 1: Debridement and proximity scrotal flap				1
56	Complication of diabetes and obesity			Time 1: Debridement and split-thickness graft		1
49	Trauma injury	Time 1: Debridement and groin flap	Time 1: Debridement and full-thickness graft from the upper arm			1
20	Complication after circumcision	Time 1: Debridement and proximity scrotal flap	Time 1: Debridement			1
			Time 2: Urethra reconstruction and full-thickness graft from the upper arm			
27	Trauma injury		Time 1: Debridement and full-thickness graft from the upper arm			1

## 4 Discussion

**Figure 2:**
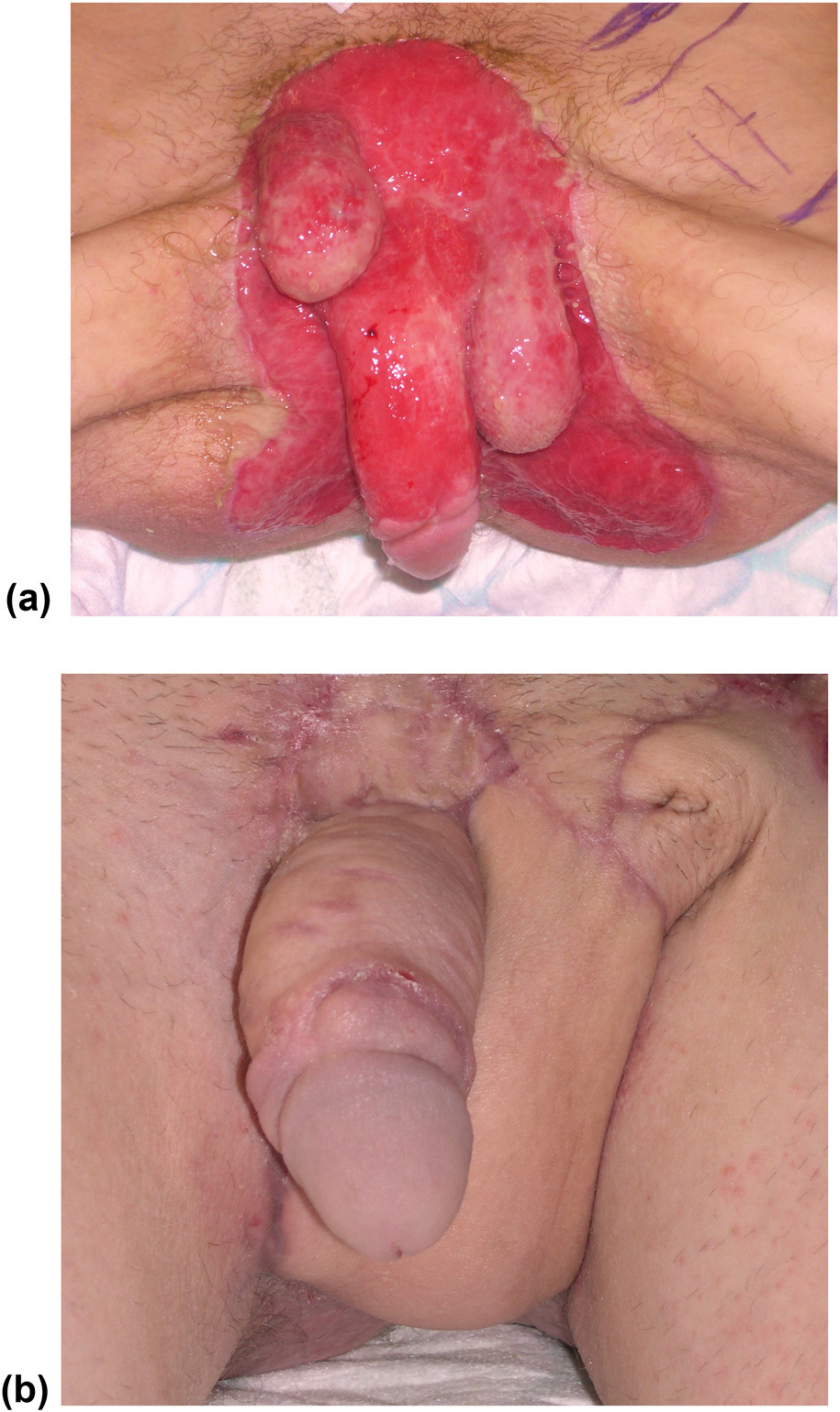
(a) Complete scrotum removal, right testicle retraction, and loss of substance of the pubic and penile region in a 45-year-old patient. (b) Reconstruction of the scrotum with inguinal flap. Reconstruction of the penis with skin graft from the inner face of the arm. Reconstruction of the perineum with a meshed graft.

Fournier’s gangrene reconstructive options are often complex and sometimes require the use of different plastic surgery techniques in a single or more times [22]. In fact, for the treatment of this condition, it is often necessary to remove necrotic and infectious tissues to reduce the risk of sepsis. Consensus on the best method for reconstruction in patients with Fournier’s gangrene has not been reached and the choice of reconstructive procedure should be based on lesions extension, patient’s characteristics, and surgeon’s experience. While skin graft is a technically simple procedure with lower donor-site morbidity compared to a flap reconstruction, graft contraction and susceptibility to trauma are a concern [23]. On the other hand, flap coverage probably provides better protection for testicular region with lower incidence of contraction, although it requires more complex and longer procedures and it is associated with higher donor-site morbidity and may be excessively bulky [24]. Moreover, the evaluation of cosmetic results is conflicting among authors [25] and objective data on patient satisfaction, function, and cosmesis are still lacking. In the cases shown, we often carried out mixed reconstruction with flap and graft. According to our experience, however, there is a very precise indication about the type of graft, whether split or full-thickness, or flap, that should be used. Both types of graft in fact have advantages and disadvantages, but, according to Thakar and Dugi, these must be compared to the specific anatomical area that has to be rebuilded [26]. First, an important consideration must be made about the timing of interventions. Following the acute inflammatory tissue phase, the exposed testicles tend to retreat and rise in the abdomen placing themselves superior to the penis (Figure 2a). Therefore, at this stage, we have always performed a surgical debridement, trying to release testicles and penis from scar granulation, avoiding healing for the second intention. This procedure may be, however, dangerous, because of the risk of devascularizing the structures during the dissection in the scar tissue. Based on our experience, the use of advanced medications such as vacuum-assisted closure therapy should be used to limit and demarcate necrotic and particularly infected areas [27], but once a grainy and bloody tissue area is obtained, it is preferable to proceed with a complex reconstruction. The use of tissue adhesives like fibrin or cyanoacrylate glue has been described in combination with skin grafts with good results [22] helping in securing skin grafts or flaps, grafting over complex contours, and decreasing the incidence of complications such as a seroma and hematoma. Nevertheless, there are no studies at this point that allow the recommendation of their use in this setting.

**Figure 3:**
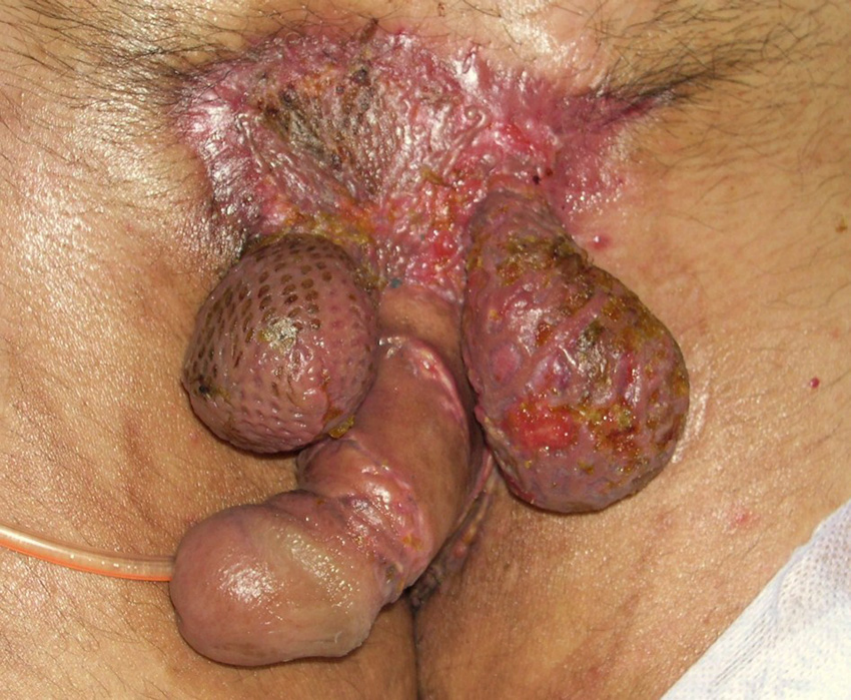
Example of using mesh grafting around the testicles and in the pubic area: unsightly and non-functional result with the risk of daily testicular traumatism.

**Figure 4:**
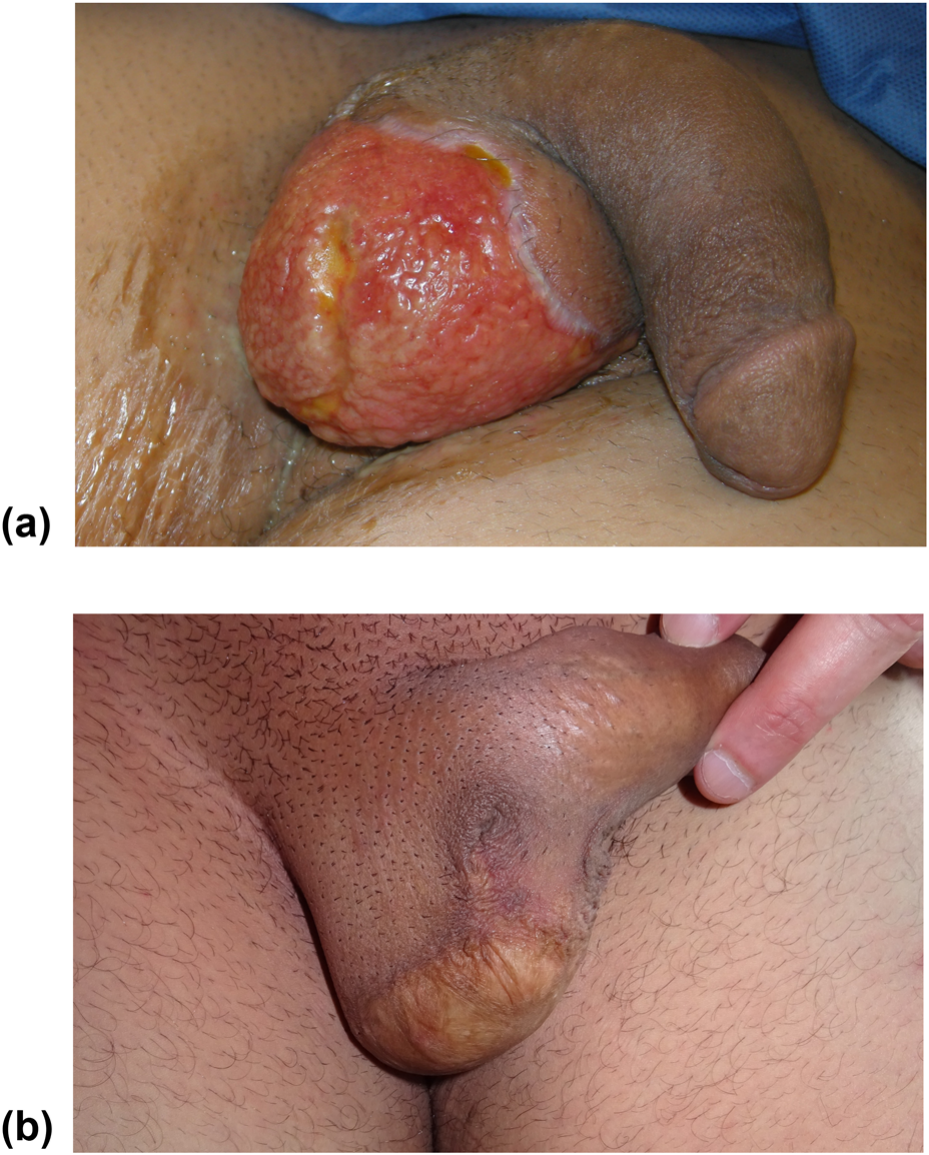
(a) Loss of substance of 2/3 of the skin of the scrotum in a 35-year-old patient with gangrene, evolved from an abscess of the gluteal region. (b) Reconstruction of the scrotum with the residual skin of the right scrotum and a sliding flap from the inner face of the contralateral thigh based on the perforating vessels of the inguinal fold.

**Figure 5:**
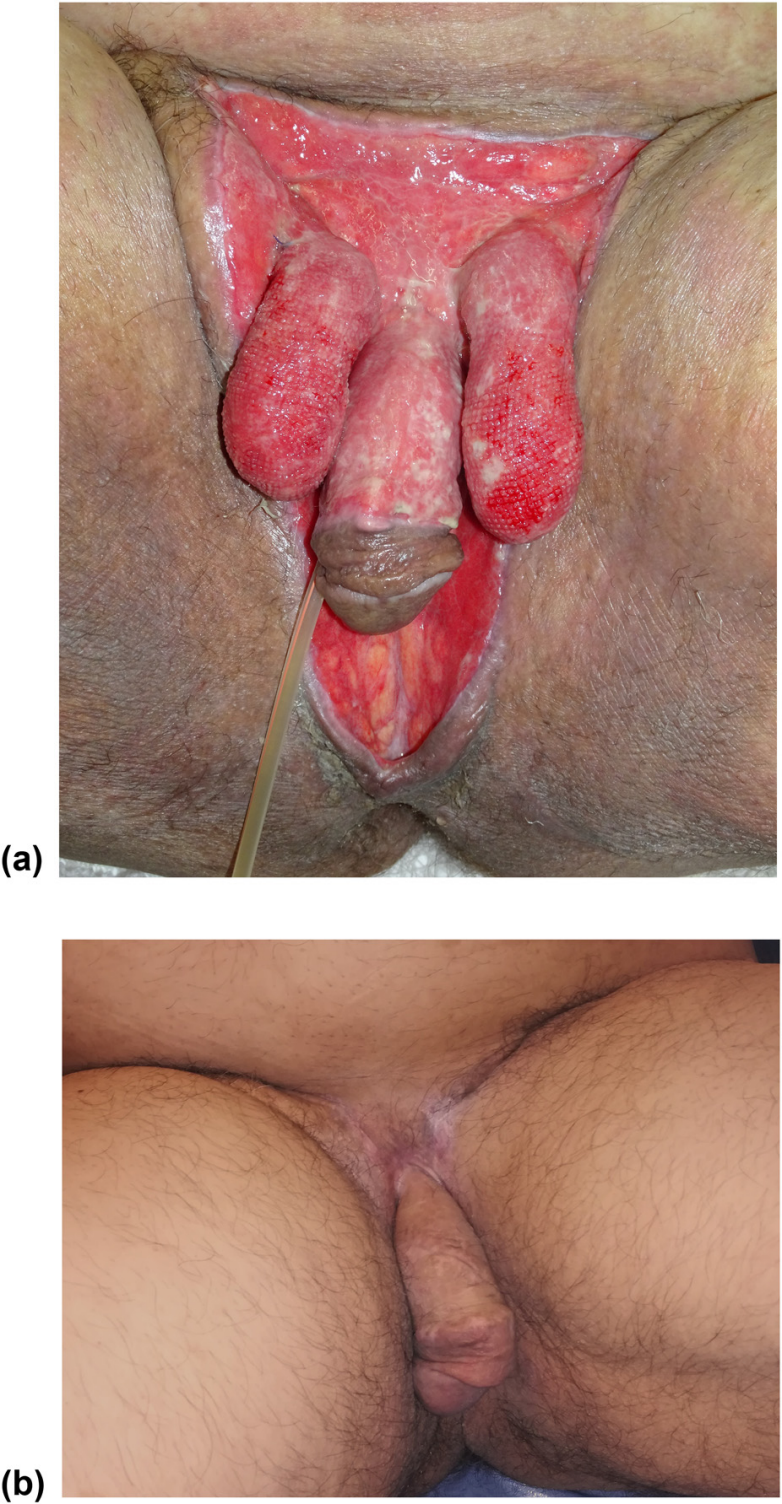
(a) A 64-year-old patient with diabetes mellitus. An important loss of substance of the pubic/perineal area, with the removal of the whole scrotum and the skin around the penis. (b) Placement of the testicles in the inner face of the thighs and skin flaps from the inner face of the thighs to close the median rafe. Graft of the skin around the penis from the inner face of the arm. The sliding flap of the abdominal skin to repair the pubis.

Once the penis and testicles are mobilized, it is possible to cover them with grafts or flaps. Patino et al. [17] documented that split-thickness skin grafts are typically used for Fournier’s cases because they are hairless, present a good evolution, cause minimal contraction, and provide good functional and cosmetic results. Moreover, the authors showed that a meshed split-thickness skin graft may represent a suitable coverage for the testicles [17], as we too have been able to experience (Figure 3). Nevertheless, we never used split skin thickness grafts as coverage for the testicles. According to our opinion, it would be preferable to cover at least part of the scrotum mobilizing the remaining parts of the scrotal area (Figure 4a and b) and, if necessary, with the skin of the groin fold when possible (Figure 2b) or by the placement of testicles in medial-inner thigh pockets (Figure 5a and b), in order to avoid a too superficial and thin covering of the testicles and a non-optimal aesthetic result (Figure 3) [28,29]. We usually performed a scrotal reconstruction with proximity flap when about more than 50% of residual scrotal tissues are preserved and if perineal tissues are available [30]. If more than 50% of scrotal tissues are compromised, we performed a groin flap [31] or testicles placement in the medial-inner tight pocket. However, this second option may determine some complications, such as thigh pouch infection, myositis, and sterility. For these reasons, we preferred the use of a groin flap in young patients and with no major complications, avoiding to perform this procedure on elderly, obese, or diabetic subjects (Figure 2b).

**Figure 6:**
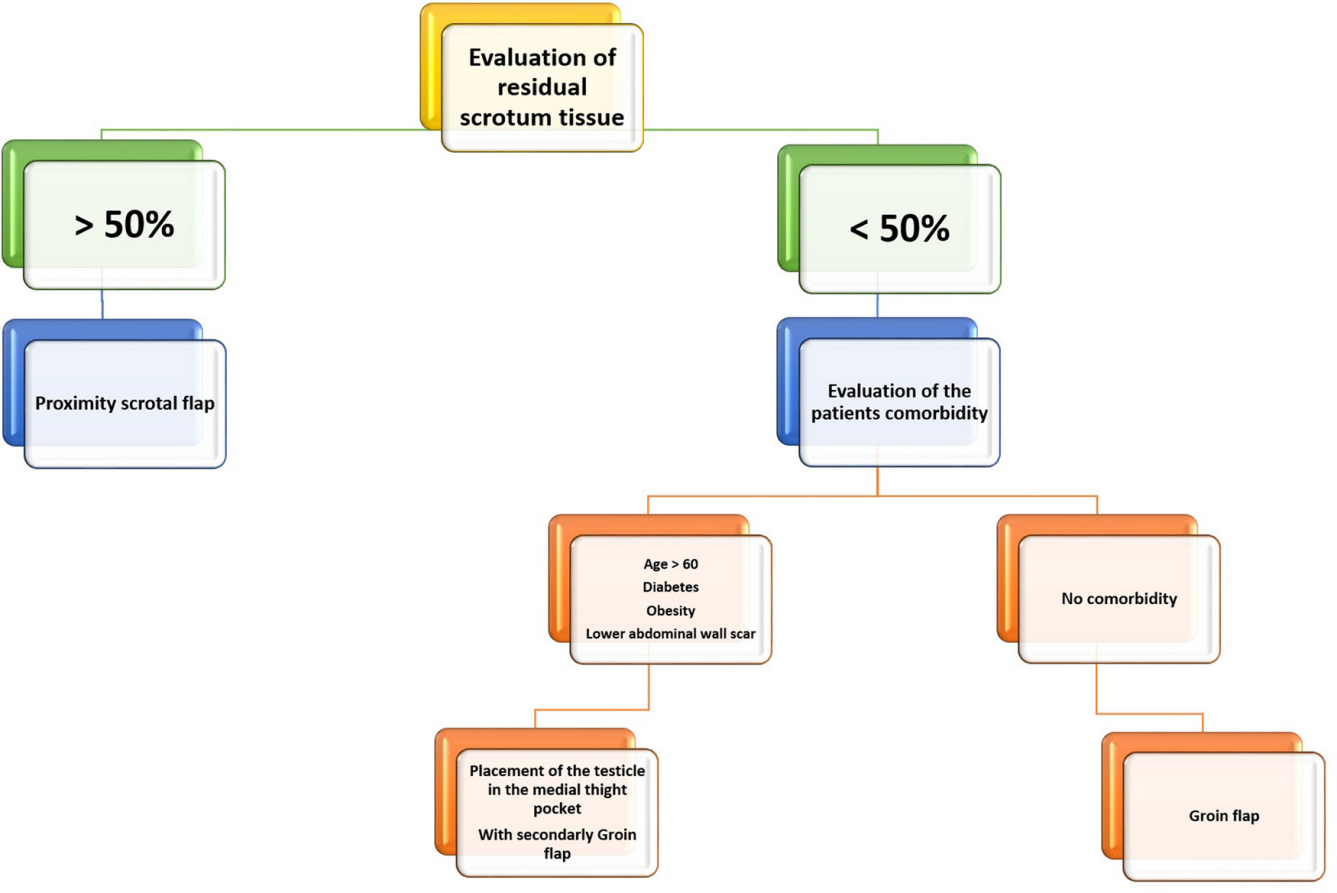
Algorithm of approach to scrotal region reconstruction treatment.

First, we proceeded to place the testicles in a medial-inner tight pocket as a temporary solution, and, secondly, we performed a reconstruction with a flap after 6 months (Figure 6). Flaps tend less to retraction with respect to grafts and accommodate the movements of the testicles avoiding compression. In ten Fournier’s disease cases, the scrotum was reconstructed with the use of proximity scrotal flap, while in the other two cases, we considered the possible direct use of a groin flap after debridement.

When the Fournier’s disease interests the penis rod, other reconstruction strategies are advisable. Thick skin grafts are recommended for their minimal contraction compared to thin skin grafts [32]. Penile skin grafting does not appear to interfere with sexual and erectile function in these cases [33]. Anandan and Mohammed asserted that when the penis region is interested by the gangrene, either split- or full-thickness skin grafts may be used, depending on preoperative evaluation by the surgeon [34]. Split-thickness grafts are used more often in genital reconstruction because graft survival rates are good, they lack hair follicles and there is no need for a local flap or subsequent grafting [35,36]. In addition, these thin skin grafts would be more similar to the penile skin that is thin and has very little subcutaneous fat. However, in comparison, thick grafts tend to provide the best durability and show reduced rates of graft penile shrinkage, secondary contraction, and fissure occurrence [34]. In this light, we always performed a full-thickness graft to cover the penis rod, considering that a too thin graft may result in a skin retraction with pain in the erection and lower elasticity and resistance to friction [37]. Using a thick graft, however, an abundance of tissue and greater elasticity may be ensured (Figure 5b). We took particular care in choosing the area of the skin graft at full thickness, and according to our observations, we used the skin of the medial face of the arms. In fact, this cutaneous zone is a humid zone, it is in continuity with a cutaneous fold and to the presence of the axillary hairs, and it is a cutaneous surface equipped with a high waterproofing [38]. It also has comparable pliability and an excellent degree of elasticity as it is in an area extremely mobile. In addition, in this way, the site of sampling of the graft is far from the diseased tissues. Despite these, any loss of substance of the perineal and perianal skin can be easily reconstructed with flaps and/or meshed skin grafts. In this case, the use of a meshed skin graft is supported by the advantage of having large amounts of skin to be taken, a greater possibility of engraftment, and a good aesthetic result. Patients underwent an average of one operating session. Such interventions were performed following a rational scheme (Figure 6) based primarily on the debridement of the area to be reconstructed and then on the reconstruction based on the evaluation of the residual healthy tissues, and using grafts or flaps of proximity. Only when the general condition of the patient had improved, we performed more complex reconstructions with pedicled flaps. We acknowledge that we did not collect data on improvement in quality of life, improvement in sexual function, or satisfaction of patients, also considering that there are no standardized data in the literature about these aspects in this cohort of patients.

Fournier’s gangrene has to be considered as a urologic urgency, with a high death rate, mainly related to the precarious patient’s health conditions. In our experience, the exposed tissues must be reconstructed as quickly as possible to avoid scarring retraction of the testicles and deformation of the penis. We hope that some valuable advice can be taken from our 16-year experience. In particular, surgical techniques have to be simple, quick, and organized based on a rational scheme, because of patients’ delicate conditions and because an excessively extended surgical approach may be complicated by a new infection. The main purpose is to rapidly cover, if possible, with less aggressive and reversible procedures, the interested areas.
